# Stepwise Catalytic Mechanism via Short-Lived Intermediate Inferred from Combined QM/MM MERP and PES Calculations on Retaining Glycosyltransferase ppGalNAcT2

**DOI:** 10.1371/journal.pcbi.1004061

**Published:** 2015-04-07

**Authors:** Tomáš Trnka, Stanislav Kozmon, Igor Tvaroška, Jaroslav Koča

**Affiliations:** 1 Central European Institute of Technology (CEITEC), Masaryk University, Brno, Czech Republic; 2 Faculty of Science—National Centre for Biomolecular Research, Masaryk University, Brno, Czech Republic; 3 On leave from the Institute of Chemistry, Slovak Academy of Sciences, Bratislava, Slovak Republic; 4 Institute of Chemistry, Slovak Academy of Sciences, Bratislava, Slovak Republic; Max Planck Institute for Biophysical Chemistry, GERMANY

## Abstract

The glycosylation of cell surface proteins plays a crucial role in a multitude of biological processes, such as cell adhesion and recognition. To understand the process of protein glycosylation, the reaction mechanisms of the participating enzymes need to be known. However, the reaction mechanism of retaining glycosyltransferases has not yet been sufficiently explained. Here we investigated the catalytic mechanism of human isoform 2 of the retaining glycosyltransferase polypeptide UDP-GalNAc transferase by coupling two different QM/MM-based approaches, namely a potential energy surface scan in two distance difference dimensions and a minimum energy reaction path optimisation using the Nudged Elastic Band method. Potential energy scan studies often suffer from inadequate sampling of reactive processes due to a predefined scan coordinate system. At the same time, path optimisation methods enable the sampling of a virtually unlimited number of dimensions, but their results cannot be unambiguously interpreted without knowledge of the potential energy surface. By combining these methods, we have been able to eliminate the most significant sources of potential errors inherent to each of these approaches. The structural model is based on the crystal structure of human isoform 2. In the QM/MM method, the QM region consists of 275 atoms, the remaining 5776 atoms were in the MM region. We found that ppGalNAcT2 catalyzes a same-face nucleophilic substitution with internal return (S_N_i). The optimized transition state for the reaction is 13.8 kcal/mol higher in energy than the reactant while the energy of the product complex is 6.7 kcal/mol lower. During the process of nucleophilic attack, a proton is synchronously transferred to the leaving phosphate. The presence of a short-lived metastable oxocarbenium intermediate is likely, as indicated by the reaction energy profiles obtained using high-level density functionals.

## Author Summary

Cell surface proteins are covered by a diverse array of glycan structures, important for mutual cell recognition and communication. These glycans are complex branched molecules assembled from monosaccharide units by a sophisticated cascade of enzymes from the group of glycosyltransferases. Disruptions in the synthesis of glycans are linked to various diseases with the most prominent example being cancer. To understand or control the process of glycosylation, the reaction mechanisms of the participating enzymes need to be known. Here we investigate the catalytic mechanism of human glycosyltransferase ppGalNAcT2 using the tools of computational chemistry. By modelling the crucial parts of the enzyme using a quantum mechanics-based description, we are able to trace the whole reaction path leading from the reactant state to the product state. Our results provide a reliable description of the motion of all important atoms during the reaction and they are fully consistent with available experimental data. The insights obtained in this study can be further used to design a potent inhibitor molecule, usable as a potential drug for diseases involving increased activity of the enzyme.

## Introduction

Protein glycosylation is known to play a pivotal role in many aspects of protein biochemistry, and there have been many examples where carbohydrate structures (glycans) carry out a significant biological function. [1–3] Glycans exist in a vast array of diverse structures built up from just a few small basic fragments. This can therefore be directly compared to the protein world, constructed purely from simple amino acids. However, in striking contrast to proteins, the structures of glycans are not encoded in any specific form analogous to the genome. [1] The so-called glycocode is just implicitly present in the regulation of hundreds of different highly specialized enzymes, glycosidases and glycosyltransferases, forming the glycosylation cascade. For this reason, understanding the reactivity of glycosyltransferases is essential to being able to decode the glycocode.

Glycosyltransferases can be divided into two main groups based on whether they invert or retain the stereochemical configuration on the anomeric carbon. The reaction mechanism of inverting glycosyltransferases is well understood and both experiments and molecular modeling support a direct displacement S_N_2-like mechanism with a protein amino acid functioning as a catalytic base. However, the same level of understanding has not yet been reached for members of the retaining group. A lot of scientific attention has been recently focused on this issue in an attempt to determine the reaction mechanism of retaining glycosyltransferases, with mixed results. [4, 5]

Throughout the group of retaining glycosyltransferases, two main mechanisms were suggested to explain the reaction. The first of them is the double-displacement mechanism, where the reaction is thought to proceed via two consecutive configuration-inverting nucleophilic substitutions, first forming a covalent enzyme-carbohydrate intermediate and then transferring the carbohydrate onto the acceptor molecule. In this mechanism, a suitably positioned amino acid residue functioning as the catalytic base is required and two enzymes, namely *α*-1,3-galactosyltransferase [6] (a3GalT) and blood-group A and B *α*-1,3-galactosyltransferase [7] were proposed to proceed with this mechanism. Theoretical calculations on truncated QM models [8] predicted this mechanism to be energetically possible. Later QM/MM calculations [9–11] also supported this mechanism.

However, there are many retaining glycosyltransferases that lack any residues that could serve as a nucleophile for the formation of the covalent intermediate. Therefore, the other possible reaction mechanism, the “internal return-like”, also called the S_N_i-like mechanism, has been suggested for these enzymes. This mechanism does not require a nucleophilic residue to be present. In this case, the reaction can proceed either as a concerted mechanism via an oxocarbenium ion pair transition state, or as a stepwise mechanism via a metastable intermediate that is subsequently captured by the acceptor nucleophile. [5] Compared with the double displacement mechanism, S_N_i substitution also seems to match the available kinetic isotope effect data. [12] The S_N_i-like mechanism was proposed for lipopolysaccharide *α*-1,4-galactosyltransferase C (LgtC) [13] and supported by theoretical studies. [4, 14] Recent experimental evidence for the retaining glycosyltransferase, trehalose-6-phosphate synthase (OtsA) [12, 15] is consistent with the S_N_i mechanism and also supports the theory that the hydrogen bond between the phosphate group and the acceptor hydroxyl plays a role in stabilizing the transition state suggested by calculations. [14] However, the existence of a short-lived intermediate remains an open question.

Recently, several QM/MM theoretical studies [10, 16, 17] have been carried out in an attempt to shed some light on this problem. All three studies used a hybrid QM/MM model of the entire enzyme and came to the same general conclusion that the S_N_i-like mechanism is the most probable one. Unfortunately, due to the substantial approximations used in these studies, many unanswered questions about the validity of their results remain.

The study on LgtC by Gómez et al. [10] used a static approach of QM/MM geometry optimisations constrained to points along a single predefined reaction coordinate, describing the difference in the lengths of the dissociating and newly forming C-O bond. Obviously, this completely neglects the second transfer process taking place at the same time—the transfer of a proton from the acceptor hydroxyl moiety onto a base represented by the leaving phosphate group. This, together with the low resolution of the scanned coordinate, led to a sudden jump of the proton upon crossing the main reaction barrier, indicated by a sharp spike in potential energy. In the end, the resulting energy profile does not describe a minimum energy path on a single potential energy surface, but a combination of two unconnected path segments corresponding to the endpoint locations of the proton.

When our manuscript was being prepared for publication, Gómez et al. published another study [17] very similar to the LgtC one, focused on the ppGalNAcT2 glycosyltransferase. Although the conclusions presented there are again in agreement with theoretical expectations and available experimental findings, the ppGalNAcT2 study shares many of the methodological problems of the LgtC one. The authors have scanned potential energy along a single predefined reaction coordinate, using a very modest quantum-chemical description of the active site, namely the Becke-Perdew pure density functional together with a small basis (SVP) and a small quantum region (80 atoms). Just the basis set itself casts serious doubts on the usability of their results, as the authors themselves show that the related error in the potential energy barrier is at least 5 kcal mol^−1^ (compared to TZVP basis), that is, about one third of the estimated barrier height. The influence of the simple density functional additionally seems to be of roughly the same magnitude. This can be related to the overall negative charge of the used QM region, as anionic systems are notoriously difficult to describe using pure density functionals due to a large self-interaction error. However, the most important shortcoming of the study in question is the fact that the authors were unable to find the transition state of the reaction, precluding any validation of the proposed reaction path.

In contrast, the study on OtsA by Ardèvol and Rovira [16] took a more rigorous approach, sampling both the nucleophilic substitution and proton transfer processes by means of two independent collective variables (CV). Their results are based on QM/MM Car-Parrinello molecular dynamics, using the metadynamics method to improve CV sampling and calculate free energy profiles, and support a single displacement with a two-step mechanism. [16] However, enhanced sampling methods like metadynamics provide correct free energy data only after the system reaches the regime of free diffusion along the reaction path. Unfortunately, computational resource constraints limit the achievable simulation lenght so severely that the free diffusion is essentially never reached. This leads to extremely noisy energy profiles, making unambiguous interpretation of the results obtained very difficult and their agreement with the expected reaction mechanism largely coincidental.

In this work, we aim to describe the reaction mechanism of a retaining glycosyltransferase as thoroughly as possible, combining the results from two different approaches in order to leverage the advantages of both while avoiding their usual shortcomings. Multidimensional energy scans are able to provide an overall view of the potential energy surface (PES), but often suffer from unsampled degrees of freedom leading to discontinuities that can pass undetected. On the other hand, minimum energy reaction path (MERP) optimization methods enable the sampling of a virtually unlimited number of dimensions, and thus guarantee that a single contiguous path will be obtained. However, there is no indication whether a given minimum energy path is the most probable and physically sound one. It can thus easily happen that a given MERP is deemed to be correct, even though an alternative path with a lower barrier exists in a different region of the PES. Such a situation is obviously impossible to detect without global information about the shape of the PES. By applying both approaches together and cross-checking the results, possible errors and artifacts can be easily identified. If the optimised MERP path is geometrically and energetically consistent with the PES, the possibility of discontinuities in the PES can be ruled out with confidence. At the same time, the shape of the PES can rule out the existence of alternative reaction pathways, validating the MERP. Unfortunately, although the idea of a combined approach is straightforward and its advantages are obvious, such a method is still not being ordinarily used to study enzymatic reactivity. Instead, studies based only on a single method with all its weaknesses are still very common.

**Fig 1:**
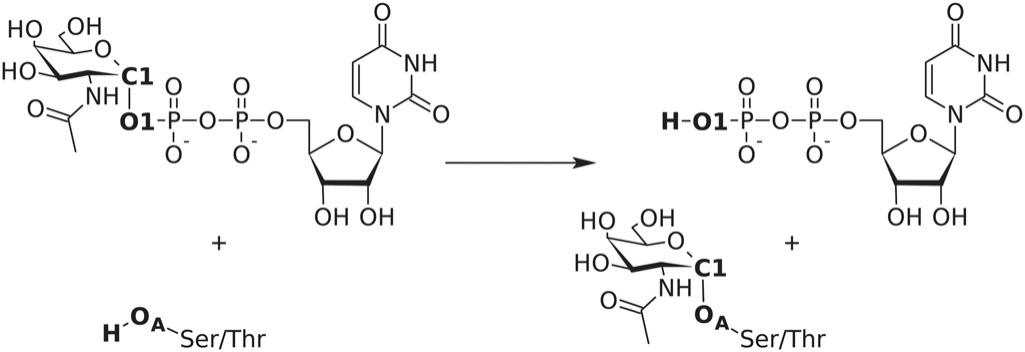
Reaction catalysed by the ppGalNAcT2 glycosyltransferase. The names of atoms used to define PES scan coordinates are set in bold.

We chose polypeptide UDP-GalNAc transferase, human isoform 2 [18] (ppGalNAcT2) as the subject of the study. This enzyme catalyses the first step in *O*-linked (mucin-type) protein glycosylation by transferring an *N*-acetylgalactosaminyl (GalNAc) group onto the serine or threonine hydroxyl moieties of an acceptor protein (Fig. 1). This glycosyltransferase exists in a large variety of isoforms exhibiting different spatial and temporal expression patterns and substrate specificities. [19] Increased activity of ppGalNAcT2 has been linked to the metastatic ability of various types of carcinoma, suggesting that targeted inhibition of a certain isoform could open the way towards selective anti-cancer drugs. [20] Detailed knowledge of the reaction mechanism and especially the transition state structure could then be used to design a potent inhibitor. Using the combined approach outlined before, we were able to obtain a reliable description of the reaction mechanism of ppGalNAcT2, including a fully optimised structure of the main transition state.

## Results/Discussion

### Reactant and product structures

The initial model was prepared from the X-ray structures of human isoform 2 [21] (PDB: 2FFU) and isoform 10 [22] (PDB: 2D7I), where the former includes a short acceptor peptide EA2 and the UDP part of the donor molecule, and the latter includes a hydrolyzed UDP-GalNAc. In both cases, the protein consists of the main catalytic domain exhibiting the common GT-A fold and a C-terminal ricin-like lectin domain in a trefoil fold. [23] Both domains are connected by a flexible linker, and as such the lectin domain does not visibly interact with the catalytic domain. Because it is also experimentally known to not be essential for catalytic activity [24], the lectin domain was cut off at conserved [25] proline 435 and not included in further studies.

The native enzyme structure contains a manganese ion in the active site, coordinating the diphosphate fragment of UDP. However, manganese usually occurs in complexes in a high-spin state possessing 5 unpaired electrons. [26] Because this fact would entail a spin-unrestricted treatment of the active site, leading to an almost twofold increase in computational cost and possible convergence problems, we opted for replacing it with magnesium. Such a change has often been used in studies of similar enzymes to allow for spin-restricted calculations, based on tests by Kóňa and Tvaroška. [27] Although the general applicability of this replacement is uncertain, experimental data on the ppGalNAc transferase isoform 1 clearly show that 89% of its activity (measured as k_cat_ using deglycosylated ovine submaxillary mucin as a poly-acceptor substrate) is retained when magnesium is used instead of manganese. [28] Computational results presented later in this work confirmed the applicability of this replacement.

The system was described by a QM/MM model, where the QM zone consisted of 275 atoms treated by density functional theory at the OPBE-D3/TZP level. Initial geometry optimisation of the model led to a dissociated carbocationic state. The reactant and product structures were subsequently obtained by pulling the anomeric carbon towards the respective oxygen atom using a restraint and then fully optimising the geometry after releasing the restraint.

### Potential energy scans

**Fig 2:**
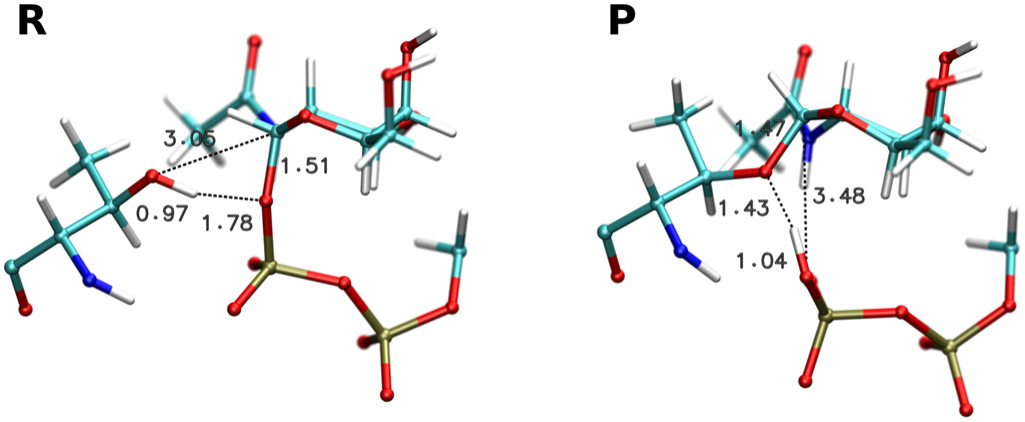
Optimized reactant and product structures.

**Fig 3:**
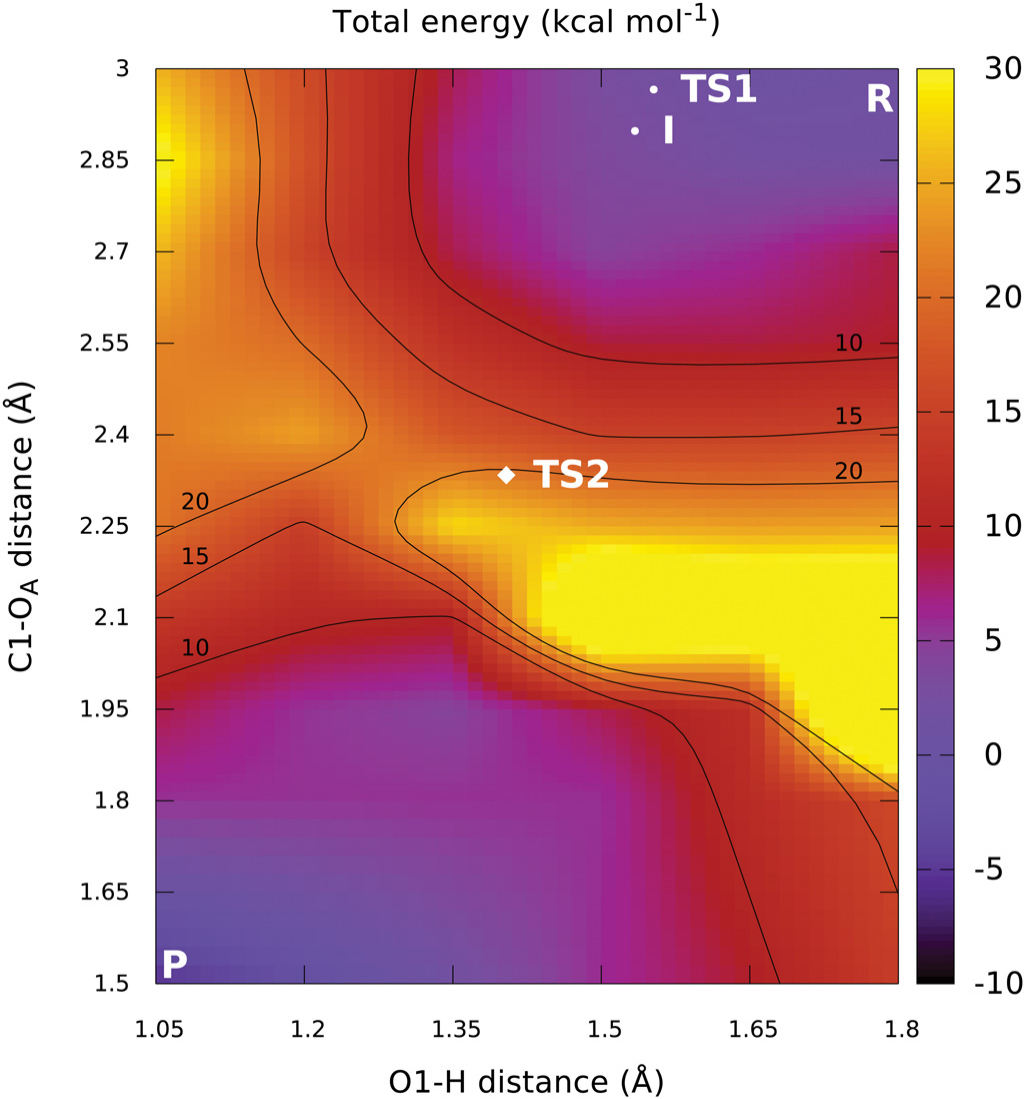
Flawed two-dimensional PES obtained by scanning distances. A serious but invisible discontinuity is present in the apparent barrier region. Additionally, the correct final projected positions of the transition states and the expected intermediate are shown for comparison.

The structures of the reactant and product complex after optimisation (Fig. 2) can be described by the parameters shown in Table 1. Based on this data, the initial 2D energy scan was done by scanning the C1-O_A_ distance from 3.00 Å to 1.50 Å and the O1-H distance from 1.80 Å to 1.05 Å, both in steps of 0.15 Å. The resulting potential energy surface map is shown in Fig. 3. It shows a large discontinuity in the location of the apparent barrier, caused by a dissociation of the GalNAc-phosphate C1-O1 bond (S3 and S4 Figs.). This implies that the calculated surface is, in fact, an artificial combination of two separate fragments of the respective 2D potential surfaces for the bound and dissociated state of UDP-GalNAc. The 2D PES region that would normally connect these two fragments is completely missing due to the inadequate sampling of the aforementioned bond dissociation process. This situation precludes any further utilisation of the results of this scan. Attempts to correct for this problem by running a three-dimensional scan (with the C1-O1 glycosidic bond length added as a third coordinate) purely in the expected transition state region failed to locate a saddle point. This leads us to conclude that even the apparent position of the reaction barrier is incorrect because of the inadequate description of the reactive processes by the chosen scan coordinates. Unfortunately, running a three-dimensional scan spanning the whole area from reactant to product is not feasible, due to the required number of scan points needed to achieve satisfactory resolution. For this reason we opted for a different set of two scan coordinates.

**Table 1:** Basic parameters of stationary point structures.

	reactant	TS1 (image 6)	intermediate (image 9)	TS2	product
*d*(C1-O_A_) (Å)	3.054	2.966	2.898	2.334	1.467
*d*(C1-O1) (Å)	1.513	2.351	2.657	3.602	3.480
*d*(O_A_-H) (Å)	0.972	1.003	1.010	1.037	1.426
*d*(O1-H) (Å)	1.780	1.553	1.534	1.404	1.038
Cremer-Pople *ϕ* (^∘^)	246.8	239.1	237.9	249.8	259.0
Cremer-Pople *θ* (^∘^)	9.1	35.3	41.9	44.4	24.3
Cremer-Pople R	0.596	0.546	0.548	0.558	0.553

**Fig 4:**
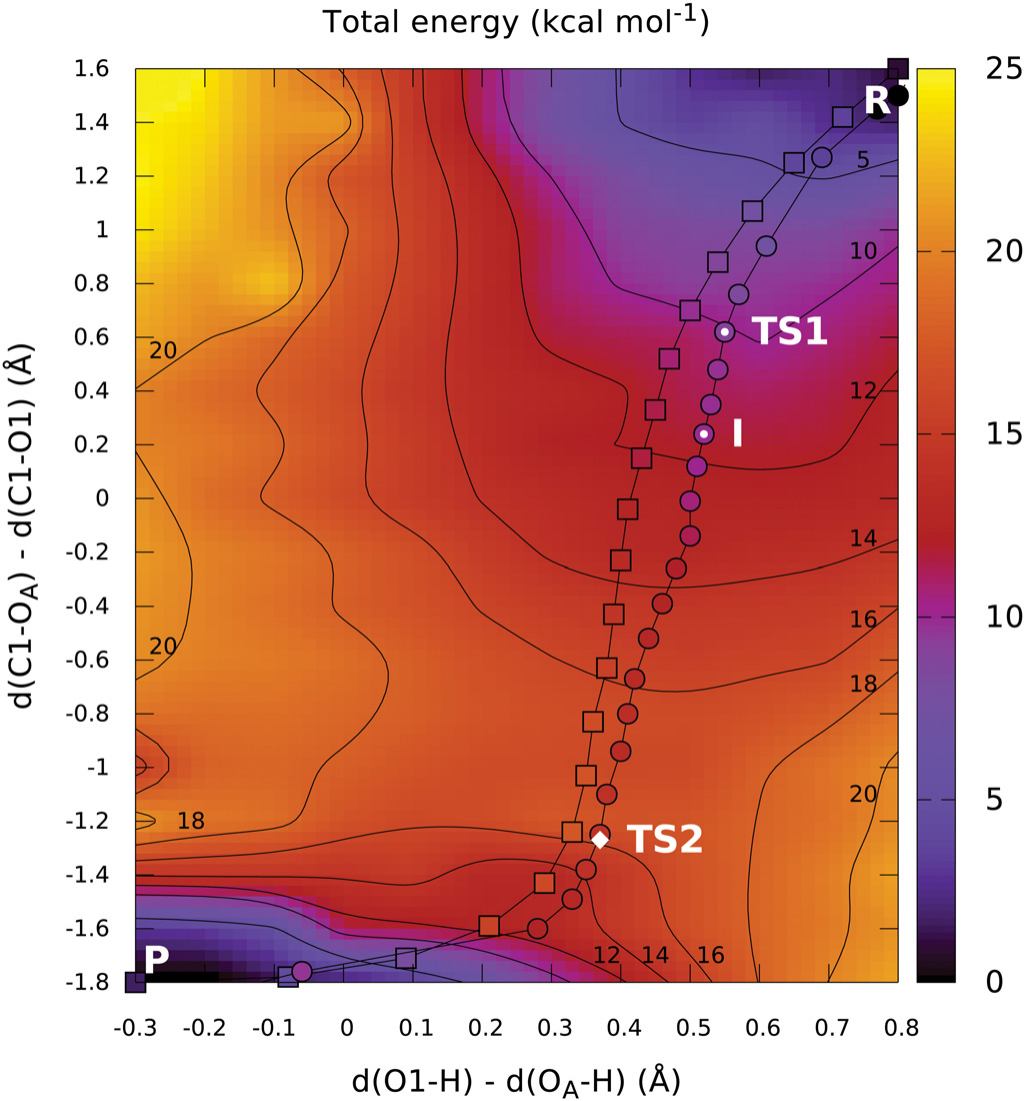
Two-dimensional PES obtained by scanning distance differences. The initial (squares) and optimized (circles) NEB paths are shown. The color of the individual path points represents the energy of the corresponding image. The position of the optimised transition state TS2 is denoted by a white diamond. The images corresponding to the estimates of two more stationary points (TS1 and intermediate) predicted by higher-level density functionals are labelled.

Coupled formation and dissociation of bonds can be described efficiently using distance difference coordinates. These are well known in the field of molecular dynamics, but they are not supported by common QM/MM software packages. After implementing them into the ADF program, we carried out another 2D energy scan, varying the nucleophilic substitution coordinate from 1.60 Å to −1.80 Å in steps of 0.20 Å, and the proton transfer coordinate from 0.80 Å to −0.30 Å in steps of 0.10 Å. This resulted in a smooth surface with no identifiable discontinuities, depicted in Fig. 4. However, several isolated data points exhibited energy significantly different from their neighbors, caused by the relatively loose geometry convergence criteria applied in order to keep the computational cost manageable. To create a clearly understandable visualisation with physically relevant contour lines, these points were removed prior to visualisation when their energy differed by more than 2 kcal mol^−1^ from the average of four directly adjacent points (for interior points) or two adjacent points along the boundary (for points on surface boundary). In total, 12 such points were removed for visualisation, amounting to only 5% of the total point count (S5 Fig.).

From the obtained PES map, we can predict a single important transition state between 16 and 18 kcal mol^−1^ above reactant energy, representing the nucleophilic attack well after dissociation of the GalNAc-phosphate bond. The extent of the saddle region delimited by the 16 and 18 kcal mol^−1^ contour lines is particularly noteworthy, as it is a clear sign of the relatively low curvature of the PES around the expected transition state. This low curvature makes direct identification of the TS candidates from the potential energy map difficult. We assume that this was the reason why attempts to optimize the transition state structure from only the scan results have been unsuccessful.

It is also clear from the potential energy map that the proton transfer cannot serve as the initiating step of the reaction, because that would lead the system into the energetically unfavorable region in the top left corner of the map. Instead, the proton is spontaneously transferred during relaxation into the product minimum, as indicated by the low-energy region in the bottom left corner and the absence of a separate proton transfer barrier in the same region.

The first phase of the reaction consists of the dissociation of the GalNAc-phosphate bond, corresponding to an increase in energy around *d*(C1-O_A_)−*d*(C1-O1) = 1.00 Å. No clear barrier can be identified for this process, as it merely appears to form a shoulder of the main reaction barrier.

### Path optimisation

NEB path optimisation from the initial approximation generated by restraint-based coordinate driving converged successfully in 100 path optimisation steps (S8 Fig.). Projection of the initial and final paths into the distance-difference 2D map are shown in Fig. 4. It is apparent that the overall path shape did not change during path relaxation. The potential energy of the individual images is depicted in Fig. 4 by the color of the path points and is clearly in reasonable agreement with the surrounding potential surface. Both facts (the consistency of the path location and the image energies) provide important evidence that the results obtained using both methods are not influenced by errors stemming from incorrect description of the reaction by the selected 2D scan coordinates or an unphysical initial path approximation.

**Fig 5:**
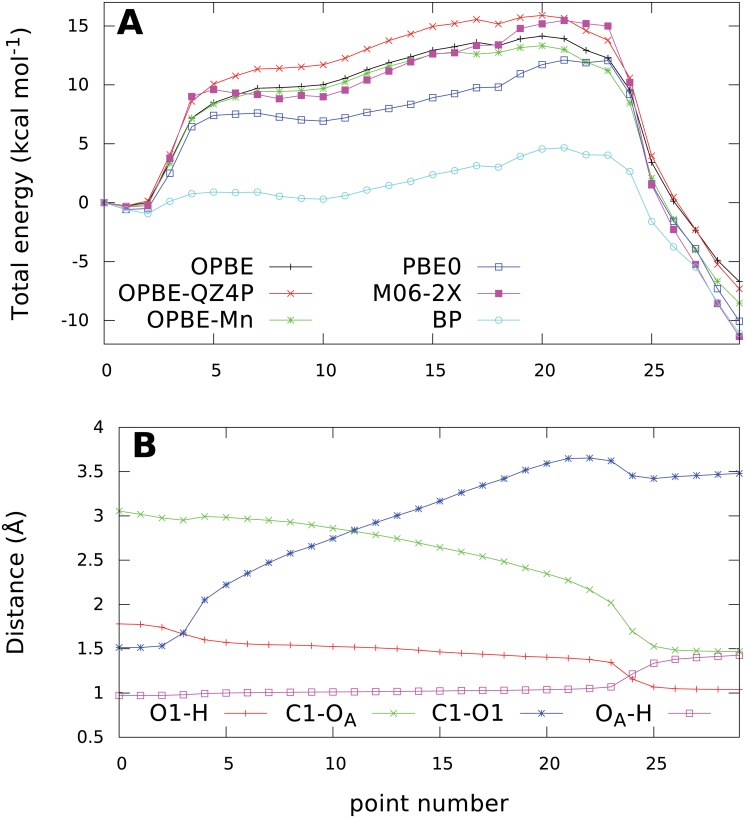
Evolution of potential energy calculated using different methods (A) and key bond lenghts (B) along the NEB path. All energies are relative to the reactant state (point 0).

The overall energy profile along the NEB path shown in Fig. 5 A again exhibits the same main features found previously in the PES. A single very large barrier is present, rising to a maximum relative energy of 14.1 kcal mol^−1^ at image 20, followed by a steep yet smooth decline to the product minimum 6.7 kcal mol^−1^ below the reactant energy.

The predicted barrier height of approx. 14 kcal mol^−1^ is in very good agreement with the phenomenological free energy barrier of approx 17 kcal mol^−1^, that can be calculated using transition state theory from the experimentally determined k_cat_ value of 3.70 s^−1^. [21] Additionally, the S_N_i mechanism observed in our study is also supported by experimental kinetic isotope effect data. [12]

To get a clearer picture of all the key processes taking place during the reaction, we can analyse the evolution of key bond lengths presented in Fig. 5B. The first phase consists of a dissociation of the C1-O1 glycosidic bond, covered by images 2–5. The distance of the attacking nucleophile does not change appreciably during this event, showing that the nucleophile is not directly involved in initiating it. On the other hand, the hydrogen bond between threonine hydrogen and phosphate oxygen shortens visibly by about 0.2 Å, as this bond is made stronger by the increased negative charge on the oxygen atom after the heterolytic cleavage of the C1-O1 bond.

The following path segments up to image 20 describe a phase of significant spatial rearrangement of the reacting species with no changes to their bonding pattern. The length of the (now dissociated) C1-O1 bond increases as the phosphate leaving group relaxes to a less strained position than the one at the start of the reaction. This gradual separation of the oxocarbenium ion—leaving group pair is probably the main reason for the gradual rise in energy, creating the nearly flat top of the barrier. At the same time, the nucleophile hydroxyl moiety is slowly inserted between the anomeric carbon and phosphate, as shown by the decreasing C1-O_A_ distance.

After crossing the saddle point region at image 20, the energy starts to decrease and from image 23 to 25, key changes in bonding take place:

- A new C1-O_A_ glycosidic bond forms between the acceptor and GalNAc, as indicated by the C1-O_A_ distance decreasing from 2.0 to 1.5 Å.
- The proton is transferred to the phosphate, while maintaining an exceptionally strong hydrogen bond to threonine with a bond length of only 1.34 Å.
- The phosphate moves back 0.3 Å closer to the GalNAc, probably thanks to the decreasing repulsion with the nucleophile oxygen.

In the last five images, the energy decreases as the system releases the conformational strain and the bond lengths relax to their equilibrium values.

To assess the impact that the approximations taken in this study might have on the validity of the calculated reaction energy profile, we calculated single-point energies on the optimized image geometries using several different methods. Replacing the magnesium ion with the natural manganese ion in high-spin configuration together with a spin-unrestricted calculation does not alter the energies significantly and the shape of the energy profile is almost entirely retained (Fig. 5A). The differences are mainly noticeable in the region of the saddle point and product minimum, where the overall barrier height is lowered by 0.8 kcal mol^−1^ to about 13.3 kcal mol^−1^. The sign and magnitude of this energy difference agrees well with the experimentally observed difference [28] in reactivity for magnesium and manganese, although considering the accuracy limits of the computational methods employed, this could be just a coincidental agreement.

Similarly, single-point energies for the profile were recomputed with a larger basis set to check for possible basis incompleteness issues. The profile calculated using the QZ4P basis [29] is qualitatively unaltered, exhibiting only a slightly increased barrier height, by 1.8 kcal mol^−1^.

Because a proper description of the reaction is wholly dependent on the performance of chosen density functional, energies were recomputed using several functionals to find possible artifacts. Although any density functional can exhibit its own share of problems, it is much less probable that a given artifact would be present in the data calculated using several different density functionals. Additionally, more complex density functionals are inherently more accurate because they are based on fewer approximations in various energy terms. For example, hybrid density functionals suffer much less from artificial locality and self-interaction error than GGA functionals, thanks to the use of the exact electron exchange term in hybrid functionals. Further improvement in accuracy is available by also including the kinetic energy density term, forming the so-called group of meta-hybrid density functionals that represent essentially the best QM methods usable for systems with hundreds of atoms. The results obtained using the hybrid PBE0 functional [30, 31] agreed well with the original OPBE ones, but a shallow minimum corresponding to a metastable oxocarbenium intermediate was now visible. The same conclusion could be drawn from a very similar profile calculated by the state-of-the-art meta-hybrid M06-2X density functional [32, 33]. This observation is similar to the findings of the OtsA study, which predicted a single displacement but a two-step reaction. [16] On the other hand, the commonly used Becke-Perdew GGA density functional [34, 35] completely failed to provide a physically sound energy profile (S7 Fig.).

**Fig 6:**
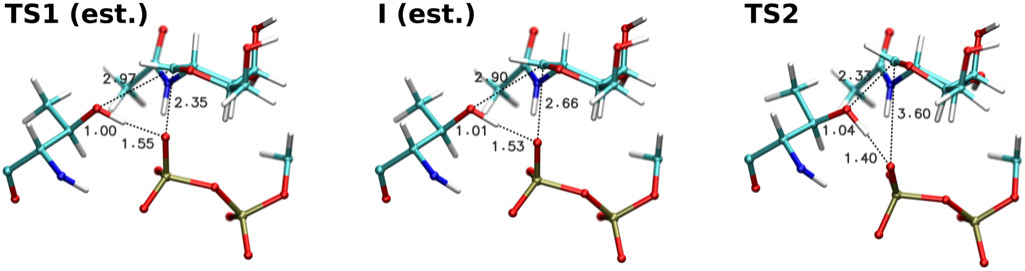
Structures of the estimated first transition state, estimated intermediate and optimized main transition state.

Unfortunately, as the minimum and thus also the preceding barrier is only present in the energy profiles calculated by hybrid density functionals, geometry optimisation of the respective stationary points would be extremely computationally demanding for a QM region consisting of 275 atoms. This is in contrast with the three confirmed stationary points that were successfully optimised using the much faster OPBE functional. For this reason, we selected image 6 as a representative structure of the first transition state and image 9 as the intermediate. Both structures are presented in Fig. 6.

Additionally, even though a minimum is present, it is only 0.7 kcal mol^−1^ deep, explaining the extremely short lifetime of the intermediate. Finally, it has to be noted that the results are based purely on potential energy data while the real physical process is controlled by free energy. The depth of the minimum may thus be significantly affected by the entropic effects included in free energy.

### Transition state optimisation

The structure of the main transition state was refined by optimising the structure of image 20 from the NEB path along the first eigenvector of an approximate numerical Hessian. After reaching convergence, the transition state depicted in Fig. 6 was obtained.

Structural changes during transition state optimisation were very small, with changes in the key distances being on the order of 0.01 Å. The final geometry of the second transition state thus has the same major features as NEB image 20. The proton is still attached to the acceptor oxygen, but at the same time it participates in a very strong hydrogen bond with the leaving group. The carbohydrate ring is in an envelope conformation with a partial half-chair character (S9 Fig.), and a new C1-O_A_ glycosidic bond is being formed. Its length in the optimised transition state is 2.33 Å, almost exactly matching the length of the dissociating C1-O1 glycosidic bond in the estimated structure of the first transition state. This observation supports the previously proposed concept [5, 12] of the two transition states involving each glycosidic bond being very similar, almost “mirror images” of each other.

To verify the correctness of the obtained transition state, a full QM/MM numerical Hessian was subsequently calculated. It contains exactly one negative eigenvalue in both the non-mass-weighted and mass-weighted (normal mode) coordinate systems, confirming that the structure corresponds to a first-order saddle point. The calculated imaginary frequency of this normal vibration mode is 96*i* cm^−1^, in line with the previously observed low curvature of the PES. Visual inspection of the normal mode motion confirmed that it represents the nucleophilic substitution process.

### Important interactions stabilizing the transition state

We have identified several interactions that probably play a key role in facilitating the reaction. These can be divided into three main groups: interactions with structural role (enforcing a proper relative positioning of the substrates), those stabilizing the positive charge on the GalNAc oxocarbenium ion and finally interactions stabilizing the negative charge on the diphosphate moiety of the UDP leaving group.

Among the structural interactions there are several hydrogen bonds coordinating the GalNAc moiety: a bond between the amidic backbone hydrogen of Gly309 and the N-acetyl carbonyl oxygen, two hydrogen bonds between Glu334 and the O4 and O6 hydrogens of GalNAc and a hydrogen bond between Arg208 and the O4 GalNAc oxygen. All of those interactions keep the GalNAc moiety rotated around the glycosidic bond towards the diphosphate group, exposing the glycosidic bond and C1 atom to a nucleophilic attack by the acceptor.

The positive charge that develops on C1 after bond dissociation interacts in a charge-dipole manner with the carbonyl group of Ala307, a member of a loop covering the *β*-face of GalNAc.

Apart from the electrostatic effects of the metal cation and hydrogen bonding with the water molecules coordinated to this ion, the leaving group is additionally forming a strong hydrogen bond with Tyr367, a hydrogen bond with Arg362 and another, relatively weak hydrogen bond with the amidic hydrogen of the acceptor threonine. There is also an important intramolecular hydrogen bond between a phosphate oxygen and the amidic hydrogen of GalNAc that further contributes to keeping the saccharide moiety suitably rotated.

However, probably the most interesting of all the active site residues is Trp331. It plays a double role: Forms a CH-*pi* interaction with the C6 hydrogen atoms of GalNAc and at the same time donates a hydrogen bond to the phosphate oxygen participating in the original glycosidic bond. This hydrogen bond is quite weak in reactant (having a length of 2.69 Å), but grows much stronger after dissociation of the glycosidic bond, achieving its minimum length of 1.76 Å around the transition state and then getting weaker again after the nucleophilic capture (2.02 Å in product).

All of the enzyme residues participating in these interactions are highly conserved and were experimentally identified as being crucial for preserving reactivity (Table 2).

**Table 2:** Parts of conserved enzyme residues included in QM region.

Residue	Included part	Function
R208	sidechain	GalNAc H-bond [64]
D224	sidechain	Mn ligand [24]
H226	sidechain	Mn ligand [24]
A307	without NH	GalNAc binding pocket [24]
G308	whole	GalNAc binding pocket [24]
G309	without CO	GalNAc binding pocket [24]
W331	sidechain	GalNAc CH-*π* [65]
E334	sidechain	GalNAc H-bond [24]
H359	sidechain	Mn ligand [24]
R362	sidechain	phosphate H-bond [25]
H365	sidechain	W331 NH-*π*
Y367	sidechain	phosphate H-bond

### Conclusions

In this study, we have shown that human isoform 2 of the polypeptide UDP-GalNAc transferase catalyses a same-face nucleophilic substitution with internal return (S_N_i). The optimized transition state for the reaction is 13.8 kcal mol^−1^ higher in energy than the reactant, while the energy of the product complex is 6.7 kcal mol^−1^ lower. This corresponds to a dissociated oxocarbenium state just before its nucleophilic capture by the acceptor threonine oxygen. During the process of nucleophilic attack, a proton is synchronously transferred to the leaving phosphate.

By coupling two different QM/MM-based approaches for investigating the reaction mechanism, namely a PES scan in two distance-difference dimensions and a MERP optimisation using the NEB method, we were able to rule out the most significant sources of potential errors. We can therefore conclude that the observations based on theoretical modeling are in good agreement with available experimental evidence [12], including the recent X-ray structures based on modified substrates. [36]

The reaction starts with a dissociation of the C1-O1 glycosidic bond of the donor UDP-GalNAc. The barrier of this step is lower than 10 kcal mol^−1^ and is thus hidden under the barrier of the rate-determining step. However, the presence of a short-lived metastable oxocarbenium ion is likely, because the corresponding energy minimum is visible in energy profiles obtained using higher-level density functionals. On the other hand, the minimum is only ca. 1 kcal mol^−1^ deep, and such a subtle energy difference is at the limit of the accuracy provided by applicable theoretical modeling approaches. Additionally, the stability of the intermediate can be affected by other entropic and environmental phenomena not considered here.

We have shown that distance-difference coordinates provide an exceedingly useful tool for the description of common reactive processes, and are certainly useful for the wider scientific community interested in mapping reaction potential energy surfaces.

Additionally, the Nudged Elastic Band method for MERP optimisation is suitable for a rapid exploration of reaction pathways, and it is immune to the coordinate sampling problems common in static energy mapping. However, knowledge of the shape of the PES is necessary to ensure that a physically relevant path is selected for optimisation.

## Methods

### QM/MM model of ppGalNAcT2

The X-ray structures of ppGalNAcT isoforms 2 (PDB ID: 2FFU) and 10 (PDB ID: 2D7I) were superimposed using Accelrys Discovery Studio Visualizer 3.1 to minimize RMS distance between corresponding C-alpha atoms of the catalytic domain (S1 Fig.). The final RMSD was 0.88 Å for C-alpha atoms and 1.33 Å for all protein atoms. Visual inspection of the active site showed near perfect overlap of the UDP molecules and neighboring side chains, allowing the coordinates of GalNAc to be directly transferred from 2D7I into 2FFU.

Afterwards, hydrogen atoms were added to the structure using the pdb2adf tool from the ADF [37] suite. First, the required fragment file for UDP-GalNAc was generated by the antechamber tool from AmberTools 1.4 [38] with partial atomic charges calculated by the AM1-BCC method [39] to give a total charge of −2. The protonation and oxidation states of relevant protein residues were assigned automatically by pdb2adf based on their chemical environment and visually checked for correctness. The protonation state of histidine 359 was manually overridden to HID as the automatically generated one (HIE) was obviously nonsensical. This residue is a ligand of the metal cofactor and therefore needs to have the N-*ε* atom unprotonated. Furthermore, water molecules present in the active site were manually rotated to create a network of hydrogen bonds where possible.

The QM region was defined to include the essential parts of the substrates and residues experimentally known to be crucial for reactivity. The UDP donor was represented by methyl diphosphate, as the ribose and uracil parts of the molecule are quite far from the reactive site. The acceptor threonine 7 of the EA2 peptide was included together with its direct neighbors, threonine 6 (excluding its amine group) and proline 8 (excluding its carboxyl group). Finally, 12 highly conserved residues interacting with the substrates were included (Table 2) as well as six water molecules that were well defined in the crystal structure (B-factors below 20). Three of these water molecules are located close to the metal ion with one of them directly serving as a ligand and the other two forming a hydrogen bond network between the first water molecule and neighboring active site residues. The other three water molecules are located approximately in the plane of the pyranose ring or slightly towards its beta-face (the face opposite to the glycosidic bond). These molecules form a hydrogen bond network, acting as hydrogen bond donors to the GalNAc O5 oxygen and the acceptor threonine oxygen. However, they are separated from the leaving diphosphate group by the carbohydrate and threonine moieties and thus cannot directly participate in the reaction as catalytic acids or bases or proton transfer mediators. The resulting system contains 252 real (non-capping) atoms in the QM region and 6051 atoms in total.

All QM/MM calculations were carried out using the Amsterdam Density Functional package [37, 40, 41] in versions 2012.01d (used only for the 2D scans and M06-2X single point calculations) and 2013.01. The NEWQMMM implementation of molecular mechanics in ADF was employed to manage the MM part of the system, described by the AMBER ff94 forcefield [42] combined with GLYCAM06 parameters [43] on the GalNAc group. The AddRemove QM/MM coupling scheme [44] was used and charges on the QM atoms were updated in every geometry iteration from the MDC decomposition [45] up to the dipole level. Hydrogen capping atoms were added by the AddRemove scheme to saturate link bonds crossing the QM/MM boundary, bringing the overall QM atom count to 275.

The QM part was described by density functional theory at the generalized gradient approximation level using the OPBE functional (a combination of the OPTX optimized exchange functional by Handy and Cohen [46] and Perdew-Burke-Ernzerhof [47, 48] correlation functional). This functional has been shown to be the best GGA functional for describing nucleophilic substitution reactions. [49] In tests, it provided results qualitatively similar to the M06-2X meta-hybrid density functional [32, 33]. The description of weak interactions was augmented by the DFT-D3 empirical dispersion correction [50, 51] in “zero-damping” form. All calculations were carried out using the all-electron Slater-type TZP basis [29] with the charge fitting set distributed with ADF.

Two types of numerical quadrature grids were employed to evaluate the electrostatic and exchange-correlation potential. A Becke grid integration scheme [52, 53] was used for all calculations using ADF 2013.01, with resolution given by the “Normal” preset. A Voronoi cell based integration method [54] was used in the energy scans and M06-2X single point calculations, because the newer Becke scheme is not available in ADF 2012.01d. The number of integration points for this method was automatically determined by ADF to meet predefined accuracy level 4 for PES scans or 6 for M06-2X calculations. To reduce random integration noise in the gradients and prevent it from spoiling the Hessian estimates, a smoothing method based on conservation of the Voronoi cells and integration points across the geometry steps was applied [53].

Because the QM/MM implementation in ADF treats MM as a perturbation to the QM system, full convergence of molecular mechanics is required in every QM geometry step. This was ensured by optimising the MM system by the scaled conjugate gradient method [55] until all MM gradient vector components decreased below 0.01 kcal mol^−1^ Å^−1^. SCF optimisation of the QM region was stopped when the maximum element of the commutator of the last two Fock matrices decreased below 10^−5^ au.

### Potential energy scans

Two-dimensional potential energy scans were carried out using two sets of scan coordinates, always starting from the optimized reactant structure. In the first scan, two distance coordinates were used: *d*(C1-O_A_) and *d*(O1-H). The second scan used two distance differences instead to provide a better description of the respective processes: *d*(C1-O_A_)−*d*(C1-O1) describing the nucleophilic substitution and *d*(O1-H)−(O_A_-H) describing the proton transfer. All scan coordinates were implemented using restraints. Support for distance difference restraints was implemented into ADF and subsequently added to the mainline distribution.

In both scans, first the respective nucleophilic substitution coordinate was scanned from the reactant value to the (approximate) product value with the second coordinate frozen. Afterwards, the second coordinate was scanned to the product value, generating the second scan dimension. The optimisation of each point started with the geometry, charges and MO coefficients of the preceding point in a given scanline and proceeded using a quasi-Newton optimizer with Broyden-Fletcher-Goldfarb-Shanno Hessian updates until the maximum gradient component decreased below 0.01 Hartree Å^−1^.

### Nudged elastic band optimisation

The minimum energy reaction path was described using the Nudged Elastic Band (NEB) approach with improved tangent estimates [56, 57]. The algorithm was based on the ASE [58] Python toolkit coupled to ADF for MM optimisation and gradient evaluation. Cartesian coordinates of all 252 real QM atoms were used to describe the configuration of each NEB image, leading to an optimisation of the reaction path in the full 756-dimensional space without any a priori assumptions regarding the reaction coordinate. Just as in the case of PES scans, the positions of all MM atoms for each image were fully optimised before every gradient calculation. The path was discretized into 30 images, where the reactant and product endpoints were kept fixed and the rest was optimized simultaneously using the FIRE algorithm [59]. This algorithm was selected because quasi-Newton algorithms do not work well with NEB, both due to the high dimensionality (28 images × 756 coordinates each = 21,168-dimensional optimisation space) and especially because the NEB Hessian matrix is not symmetric [60] and therefore can not describe the locally quadratic surface assumed by quasi-Newton algorithms. The FIRE algorithm is the most sophisticated algorithm implemented in ASE that is able to deal with this problem. All internal optimizer parameters were kept at their default values.

We observed a significant instability of the pure NEB path in regions where the PES is relatively flat (especially in the reactant and product basins), leading to the formation of “kinks” in the path and subsequent divergent lengthening of the affected path segments. A partial perpendicular force term [56] was added to keep the path stable and smooth. The fraction of perpendicular force included was determined by

$$\begin{array}{c} f\left(\Phi \right)=\frac{1}{2}\left(1+\cos\left(\pi \left(\cos\Phi \right)\right)\right) \end{array}$$

where *ϕ* denotes the angle between adjacent path segments. Although this means that the result is no longer a rigorously correct minimum potential energy path, but just an approximation of it, the difference is minimal, and it is only visible in regions that are not particularly interesting in terms of the reaction mechanism. The force constant *k* for NEB springs was set to 5 eV Å^−1^. Path optimisation was stopped when the maximum element of total NEB gradient decreased below 0.0025 Hartree Å^−1^.

Initial approximation of the minimum energy reaction path was created by manually picking 5 points (pairs of distance difference values) from the potential energy surface and applying spline interpolation to obtain 21 distance difference pairs uniformly spaced along the curve. Structures were then generated by successive optimisation starting from the reactant structure with the two distance difference coordinates restrained to the values corresponding to a given point. All these structures were directly used as NEB images. Because the NEB approach outlined above forces the images to be equidistant with respect to their 756-D Euclidean distance, additional images were added by linear interpolation between the obtained structures until all path segments were shorter than 0.25 Å. To keep the implementation simple, only the positions of QM atoms were interpolated; the positions of MM atoms and the QM charges were directly copied from the nearest parent structure.

### Transition state refinement

The final transition state structure was obtained by optimising image 20 of the converged NEB path using a quasi-Newton optimizer with Bofill’s Hessian update scheme [61] and a gradient convergence criterion of 0.001 Hartree Å^−1^. Full numerical Hessian from a preceding calculation on image 23 was used as the initial Hessian for the optimisation. After reaching convergence, a full numerical Hessian of the total QM/MM energy was calculated for the optimized structure using symmetric central two-point finite differentiation of gradients with a step of 0.01 Å.

Cremer-Pople conformational parameters [62] for the carbohydrate ring were calculated using the cp.py script by Hill and Reilly [63].

## Supporting Information

S1 Fig Superposition of the crystal structures used to build the initial model of ppGalNAcT2. Isoform 2 (PDB ID 2FFU) is shown in gray and isoform 10 (PDB ID 2D7I) in green. The ricin-like lectin domains (left part) were not considered in this study. The overlapping crystal positions of the uridine diphosphate and the positions of the EA2 acceptor peptide (from 2FFU) and GalNAc (from 2D7I) are depicted in ball-and-stick representation.
(TIF)

S2 Fig QM and MM energy components of the distance-based 2D PES scan surface. (EPS)

S3 Fig Values of two important bond distances not sampled in the distance-based 2D scan. Note especially the abrupt change of the C1-O1 distance upon crossing the apparent enery barrier. The positions of the expected stationary points obtained by the path optimisation visibly fall into a region where both distances shown here still have reactant-like values, confirming the unphysical nature of distance-based scan results.
(EPS)

S4 Fig Projection of the 2D distance-based scan points into the distance-difference coordinate system. A discontinuity is visible in the region between both transition states. It is clear that the points obtained by scanning the two distances fall either into the reactant or product basin and don’t sample the barrier region at all.
(EPS)

S5 Fig Raw data points obtained by scanning the two distance-difference coordinates. Twelve outlying points (crossed out) were not used for visualisation of the total energy surface in order to obtain physically meaningful contour lines.
(EPS)

S6 Fig QM and MM energy components of the 2D PES scan surface obtained using distance differences. (EPS)

S7 Fig QM and MM energy components for the images along the optimized reaction path. Notice the shallow minimum in QM energy around image 9 (expected intermediate), and a corresponding maximum in MM energy in the same region. These two contributions cancel out (for the OPBE functional), leading to no minimum being present on the total energy profile. QM energy profile calculated using the Becke-Perdew functional is included, illustrating that this method is unable to produce a physically meaningful description of the reaction.
(EPS)

S8 Fig Evolution of total energy profiles along the NEB path during the course of path optimisation. Changes between iterations 80 and 100 (final) are negligible, confirming that the path is well-converged.
(EPS)

S9 Fig The conformational itinerary of GalNAc during the reaction. The third quadrant of the pseudorotational diagram constructed from Cremer-Pople ring puckering coordinates is shown.
(EPS)

S1 Supporting Information Complete structures of all (optimised or estimated) stationary points. Two files are provided for each stationary point—a PDB file for the whole system and a XYZ file with only the QM region.
(ZIP)
