# Supplementary figures and images for: Stepwise Catalytic Mechanism via Short-Lived Intermediate Inferred from Combined QM/MM MERP and PES Calculations on Retaining Glycosyltransferase ppGalNAcT2
Source: PLoS Comput Biol. 2015 Apr 7;11(4):e1004061. doi: 10.1371/journal.pcbi.1004061 (PMC4388629; doi:10.1371/journal.pcbi.1004061)

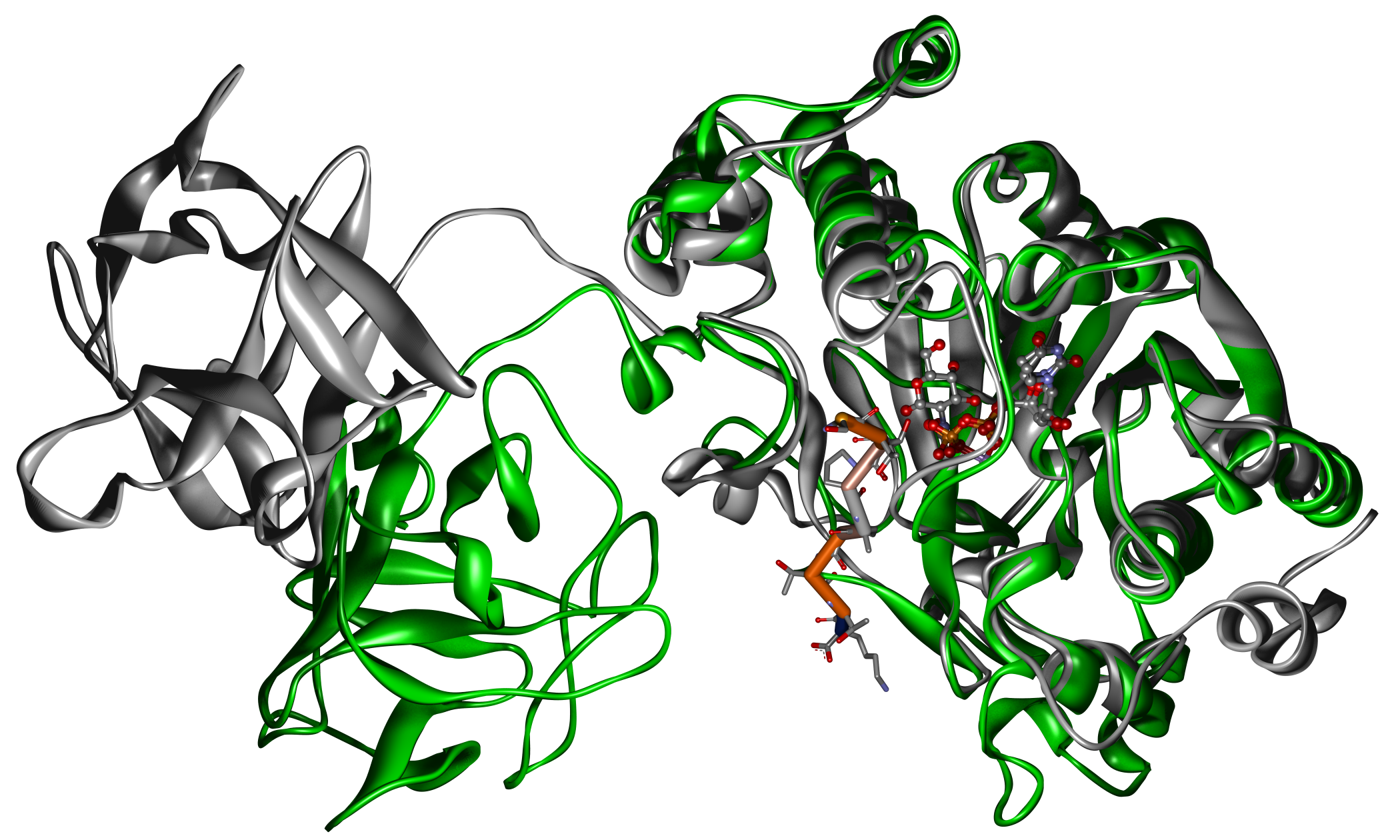

Supplement: S1 Fig — Isoform 2 (PDB ID 2FFU) is shown in gray and isoform 10 (PDB ID 2D7I) in green. The ricin-like lectin domains (left part) were not considered in this study. The overlapping crystal positions of the uridine diphosphate and the positions of the EA2 acceptor peptide (from 2FFU) and GalNAc (from 2D7I) are depicted in ball-and-stick representation. (TIF) [file pcbi.1004061.s001.tif]
